# Influence of biophysical properties on temporal filters in a sensory neuron

**DOI:** 10.1186/1471-2202-14-S1-P347

**Published:** 2013-07-08

**Authors:** Jan-Hendrik Schleimer, Susanne Schreiber

**Affiliations:** 1 Bernstein Center for Computational Neuroscience, Berlin, Germany; 2 Institute for Theoretical Biology, Humboldt University, Berlin, Germany

## 

Sensory pathways implement filters that extract relevant information from the environment [1]. These filtering properties depend in intricate ways on the biophysical parameters of the underlying neuronal architecture. Understanding the link between computational aspects, such as response properties or spike statistics, and the underlying biophysics is a question that can be addressed with theoretical methods.

Many primary sensory neurons operate in a mean-driven regime, where mean intensity is represented by the average firing rate, while the temporal structure of the stimulus around its mean is encoded into the particular structure of the spike train in analogy to an irregular sampler [2]. Examples of neurons applying this strategy include the paddlefish's electrosensory system [3], grasshopper auditory receptors [4], or the vestibular system of the turtle [5].

Spikes are generated from the dynamics of voltage-dependent ion channels. Different sensory neurons, however, may use different compositions of spike-generating channel types. These do not only render the cell excitable in the first place, they also have a large influence on a neuron's transfer function. In addition, some ion channels shape the subthreshold dynamics of neurons, but it is less clear, how they affect the temporal structure of spike trains when the neurons are driven tonically.

Several channel types, including HCN channels, are known to cause subthreshold resonance. This property results in largest subthreshold response amplitudes of a neuron at a particular frequency, hence termed the resonance frequency. The resulting band-pass like filter properties have a strong effect on fluctuation-driven neurons. Their influence on tonic spiking, however, has been less explored. Accordingly, the influence of channels that cause subthreshold resonance, such as HCN, on sensory systems like the hair cells of the mouse inner ear is still under debate [6]. Here, we use phase-response-curve-based methods within the framework of numerical continuation to resolve whether and how the effects of these channels carry over into the suprathreshold, mean-driven dynamics.

To this end, we use computational models of hair-cell mechanoreceptors [7]. Our approach is to, first, derive the phase oscillator that is input-output equivalent to the full biophysical model. Then, applying linear response theory, the transfer function can be estimated in the regime of weak-amplitude stimulation [8]. This transfer function maps time-dependent stimuli to the instantaneous firing rate. We show that the poles and zeros of the transfer spectrum are related to the Fourier components of the systems phase response curve (PRC) as well as the average intrinsic noise level, which is due to a finite ensemble of stochastic ion channels.

We find that both properties (1) the PRC - a consequence of the deterministic part of the dynamical system - and (2) the intrinsic stochasticity, are affected by the presence of HCN channels. Further, we use an overall measure of information transmission, the stimulus-response information, to approximate the contribution of HCN channels to information transfer in given frequency bands.
